# Longitudinal amyloid burden with combined [^11^C]PiB and [^18^F]NAV4694 PET scans

**DOI:** 10.1162/IMAG.a.1148

**Published:** 2026-03-09

**Authors:** Brecca Bettcher, Max McLachlan, Matthew Zammit, Andrew McVea, Alexandra H. DiFilippo, Dhanabalan Murali, Ali Pirasteh, Erin Jonaitis, Rebecca Landhough, Tobey Betthauser, Sterling Johnson, Bradley T. Christian

**Affiliations:** University of Wisconsin-Madison School of Medicine and Public Health and Waisman Center, Madison, WI, United States

**Keywords:** PET, Alzheimer’s disease, amyloid, longitudinal harmonization, [^11^C]PiB, [^18^F]NAV4694

## Abstract

The β-amyloid radiotracer [^18^F]NAV4694 is a desirable alternative to [^11^C]PiB, possessing similar imaging characteristics and favorable radiopharmaceutical distribution. This work examined the consistency of amyloid measures between [^11^C]PiB and [^18^F]NAV4694 as participants transition between radiotracers in longitudinal studies. Thirty-five participants with ≥1 [^11^C]PiB scans, followed by a [^18^F]NAV4694 scan, were recruited from ongoing AD studies at the University of Wisconsin. Amyloid stability was evaluated in Aβ- individuals and amyloid accumulation was evaluated in Aβ+ individuals. In Aβ- participants, [^18^F]NAV4694 measures were consistent with the preceding [^11^C]PiB measures, showing no significant differences in CL values. Aβ+ participants exhibited an average annualized Aβ accumulation of 6.0 ± 1.8 CL/yr, consistent with modeled [^11^C]PiB Aβ projected outcomes. [^18^F]NAV4694 demonstrated both consistency in trending amyloid accumulation and constancy in sustained Aβ- participants compared with [^11^C]PiB. This study highlights how both radiotracers can be integrated within a single analytical framework under a uniform processing pipeline.

## 1 Introduction

[^11^C]PiB PET imaging provided the earliest evidence for the in vivo detection of neuropathology related to Alzheimer’s disease (Klunk et al., 2004). Regarded as the gold standard PET radiotracer for beta-amyloid (Aβ) imaging, [^11^C]PiB binds to Aβ plaques with high affinity and specificity (Cohen et al., 2012; Klunk et al., 2004). [^11^C]PiB demonstrates robust cortical binding during the preclinical disease stage with high predictive value for related neuropathology and AD progression (Jack, Knopman, et al., 2013; Sperling et al., 2011; Villemagne et al., 2013). The relatively short radioactive half-life of [^11^C]PiB (^11^C t_1_/_2_ = 20.3 minutes) limits its use to facilities with on-site cyclotrons. The unparalleled scientific value of Aβ-PET imaging has driven the demand for the development of alternative radiotracers using longer-lived isotopes that are amenable to regional distribution across research sites.

Amyloid radiotracers labeled with ^18^F (t_1/2_ = 109.8 minutes) have addressed this demand, accommodating multiple injections per manufactured batch and a broader geographical distribution for PET imaging. In 2012, [^18^F]AV-45 (florbetapir) became the first FDA-approved [^18^F] Aβ-radiotracer for human use, with FDA approval of [^18^F]flutemetamol and [^18^F]florbetaben following shortly thereafter (Yang et al., 2012). The proliferation of Aβ-radiotracers has added to the variability of amyloid quantification and created a need for scaling outcome measures of Aβ burden across multicenter studies. The large international working group, Global Alzheimer’s Association Interactive Network (GAAIN), has addressed the standardized measurement of Aβ binding across radiotracers through the Centiloid scale (CL) (Klunk et al., 2015). The CL scale is a linear scale, with an average distribution centered at 0 corresponding to Aβ plaque-free young controls and 100 corresponding to mild–moderate severity of AD (Klunk et al., 2015). Established linear equations that convert SUVr to CL as well as validation sets are available for radiotracers [^11^C]PiB, [^18^F]NAV4694, [^18^F]florbetaben, [^18^F]flutemetamol, and [^18^F]florbetapir (Battle et al., 2018; Klunk et al., 2015; Navitsky et al., 2018; Rowe et al., 2016, 2017). The methods for CL quantification, including the reference region, have been adapted to accommodate the specific characteristics of the selected radiotracer (Landau et al., 2013; McLachlan et al., 2025).

The Aβ-radiotracer [^18^F]NAV4694, formerly known as [^18^F]AZD4694, is a desirable alternative to [^11^C]PiB, possessing highly similar imaging characteristics, steric structure, in vivo kinetics, and dynamic signal range (Rowe et al., 2013, 2016). Head-to-head [^11^C]PiB and [^18^F]NAV4694 studies have reported high neocortical SUVr linear correlations (Rowe et al., 2016). Relative to other ^18^F-labeled amyloid radiotracers, [^18^F]NAV4694 has demonstrated lower white matter retention and higher cortical gray matter binding. The variance ratio provides a measure of variability for an alternative radiotracer relative to [^11^C]PiB, and is defined as the standard deviation of CL values obtained with an ^18^F-radiotracer divided by the standard deviation of CL values of [¹¹C]PiB in the same cohort of young healthy individuals. Reported CL variance ratios relative to [^11^C]PiB for alternative ^18^F amyloid tracers include [^18^F]flutemetamol (1.19), [^18^F]florbetaben (1.96), and [^18^F]florbetapir (4.62) (Battle et al., 2018; Navitsky et al., 2016; Rowe et al., 2017). [^18^F]NAV4694 exhibits the lowest variance ratio of 0.85 (Rowe et al., 2016). Broadly, [^18^F]NAV4694 most closely mirrors the performance of [^11^C]PiB, grounding it as the preferred alternative among the current ^18^F-Aβ-radiotracers.

Longitudinal studies embracing new technologies, including novel radiotracers, are challenged to preserve biological measures while removing technical variability. In longitudinal Aβ PET imaging studies that transition from [^11^C]PiB to an alternative Aβ-radiotracer, the interpretation of longitudinal changes under conditions of aggregated data across radiotracers must account for sources of variability. The strong similarities between [¹¹C]PiB and [^18^F]NAV4694 present an opportunity for both radiotracers to utilize the same processing pipeline with minimal technical variability. Participants from a large longitudinal cohort receiving [^11^C]PiB scans underwent a terminal [^18^F]NAV4694 amyloid PET assessment, offering an opportunity to examine the transition between radiotracers in a well-characterized dataset. In this work we examine the consistency of [^18^F]NAV4694 amyloid measures with respect to [^11^C]PiB as participants transition from [^11^C]PiB to [^18^F]NAV4694 in longitudinal studies. This objective was addressed by assessing amyloid stability in an Aβ- group and the rate of amyloid accumulation in an Aβ+ group with respect to longitudinal [^11^C]PiB amyloid.

## 2 Methods

### 2.1 Study participants and PET scan history

Participants were recruited from ongoing longitudinal studies at the University of Wisconsin-Madison including the Wisconsin Alzheimer’s Disease Research Center (ADRC) and the Wisconsin Registry for Alzheimer’s Prevention (WRAP) study, two cohorts enriched with participants with a familial history of AD (Johnson et al., 2018). Inclusion criteria for this study included participants with one or more [^11^C]PiB PET scans, followed by a [^18^F]NAV4694 PET. Across all participants, the [^18^F]NAV4694 PET scan represented the terminal amyloid PET scan. No additional exclusion criteria were imposed beyond those specified for WRAP and ADRC study enrollment (Johnson et al., 2018). Accordingly, individuals with other medical conditions or comorbidities were not excluded. All participants received structural T1-weighted MR imaging. Participants’ written consent was obtained according to the Declaration of Helsinki and under IRB approval.

### 2.2 Imaging procedures

T1-weighted MR images were acquired using an inversion recovery fast spoiled gradient-echo (IR-FSPGR) pulse sequence on a 3.0-T GE Signa 750 MRI system (Signa MR750, GE Healthcare) to provide high-resolution structural information for image registration and spatial normalization. PET imaging with [^11^C]PiB or [^18^F]NAV4694 was conducted on a Siemens ECAT EXACT HR+ PET scanner or a Siemens Biograph Horizon PET/CT scanner. Participants were permitted to change scanners across observations. Participants received a target dose of 15mCi of [^11^C]PiB and 8mCi of [^18^F]NAV4694 injected intravenously with a bolus infusion. PET imaging was performed for 20 minutes from 50 to 70 minutes post-injection for both radiotracers. PET data were reconstructed, corrected for attenuation, deadtime, scatter, and radioactive decay, and binned into four 5-minute frames.

### 2.3 Amyloid PET outcome

Amyloid measures were calculated following the standard Centiloid (CL) method as described by the literature (Klunk et al., 2015). Briefly, participant-specific T1-weighted MRIs were coregistered to the Montreal Neurological Institute 152 (MNI-152) template. Each average PET image was coregistered to its participant-specific MRI and subsequently normalized into MNI152 space using the transformation derived from the MRI unified segmentation method. Image processing was performed with SPM8. No smoothing was applied. SUVR images were generated by voxel normalization to the mean activity in the whole cerebellum, and the mean cortical SUVR was extracted from the Centiloid cortex ROI. The target and reference ROIs were the same across radiotracers. The following equations were implemented to convert SUVr to CL: CL_PIB_ = 100 × (SUVR_PIB_ – 1.009)/1.067, CL_NAV4694_ = 100 × (SUVR_NAV4694_ – 1.031)/1.172). To validate the CL pipeline, a “Level-1” analysis was performed with the published [^11^C]PiB dataset obtained from GAAIN. A threshold of 18 CL was used to discriminate individuals who are amyloid positive (Aβ+) as implemented previously (Zammit, Bruzzone, et al., 2025).

### 2.4 Statistical analysis

The goal of this analysis was to compare the changes in Aβ burden from consecutive [^11^C]PiB observations, referred to as PiB-PiB, against [^11^C]PiB to [^18^F]NAV4694 observations, referred to as PiB-NAV. This objective was addressed by assessing CL stability in an Aβ- group and the rate of CL increase as a measure of amyloid accumulation in an Aβ+ group.

#### 2.4.1 Aβ- group

Amyloid stability across radiotracers was evaluated in firmly amyloid-negative individuals with a terminal PET scan <10 CL. A conservative threshold of 10 CL was imposed on the terminal [^18^F]NAV4694 observation to best reflect the absence of neuritic plaque and help mitigate capturing low levels of amyloid accumulation in early disease progression (Amadoru et al., 2020; Collij et al., 2024). No threshold was required for the previous [^11^C]PiB scans. This amyloid-negative subset was used as a proxy for test–retest stability assessment. For each transition between subsequent scans, raw CL change (ΔCL) and annualized CL change (ΔCL/Δt) were computed. An individual participant thus had two change scores for every sequential pair of amyloid scans: raw CL change PiB-PiB or PiB-NAV and annualized CL change PiB-PiB or PiB-NAV. A Wilcoxon rank-sum test assessed whether change between all PiB-PiB vs PiB-NAV scans was similar under the two conditions. Participants with a terminal PET scan <10 CL and at least two prior [¹¹C]PiB scans were selected for a within-subject comparison of PiB-PiB and PiB-NAV changes, with raw and annualized CL change scores analyzed using the Wilcoxon signed-rank test. For participants with three or more [^11^C]PiB scans, a Friedman test was conducted for multiple comparisons. Rank tests were two-tailed with p < 0.05 considered significant. Effects of radiotracer in the subset were evaluated using a longitudinal linear mixed effect model including fixed effects of age, radiotracer, and scanner as well as subject-specific random intercepts (Model: CL ~ Age + Tracer + Scanner + (1|Participant)). Statistical analyses were performed using R Statistical Software R version 4.3.2.

#### 2.4.2 Aβ+ group

Annualized CL change from [^11^C]PiB to [^18^F]NAV4694 in Aβ+ individuals was evaluated against [^11^C]PiB accumulation rates modeled using Sampled Iterative Local Approximation (SILA) (Betthauser et al., 2022; Zammit et al., 2023). SILA is a temporal modeling method validated for amyloid PET, providing a longitudinal time course of amyloid accumulation, including individualized estimates of amyloid age of since amyloid onset. Amyloid age, also known as AD time, provides an estimate of how many years an individual has been amyloid positive. An amyloid age of 0 corresponds to the time of reaching the positivity threshold. This metric offers a way to compare the level of neuropathology on an individual basis. Due to the small number of participants with multiple positive [^11^C]PiB scans in this sample, an estimated [^11^C]PiB accumulation rate was generated from a larger sample in this cohort. A [^11^C]PiB SILA model was generated from 172 [^11^C]PiB participants participating in the WRAP study. The rate of amyloid accumulation for the full sample was calculated from an amyloid age of 0–15 years and compared against the annualized CL change from [^11^C]PiB to [^18^F]NAV4694 in Aβ+ individuals.

## 3 Results

### 3.1 Visualization of longitudinal Aβ change

A total of 35 participants met inclusion criteria with participants receiving up to five [^11^C]PiB scans over a duration of up to 14 years. The average duration between the last [^11^C]PiB scan and following [^18^F]NAV4694 scan was 3.1 years (range 1.9–10.5 years). In the 27 participants who received consecutive [^11^C]PiB scans, the average duration between the last two [^11^C]PiB scans was 3.3 years (range: 2.2–8.9 years). Over the course of the study, 15 participants changed scanners across observations, whereas 20 participants were imaged consistently on the same scanner. Participant details are outlined in Table 1.

**Table 1.** Participants were recruited from WRAP and ADRC longitudinal studies at UW-Madison (selected from >2,000 participants).

**Participants (n)**	**35**
Age, years (at last observation)	73 ± 7
Female (%)	28 (80%)
APOE e4 carrier (%)	8 (23%)
**Total [^11^C]PiB scans**	**103**
Participants with 1 PiB scans	8
Participants with 2 PiB scans	5
Participants with 3 PiB scans	6
Participants with 4 PiB scans	13
Participants with 5 PiB scans	3
**Total [^18^F]NAV4694 scans**	**35**
Aβ > 18 CL	13
10 CL < Aβ < 18 CL	4
Aβ < 10 CL	18
**Average duration between scans in years**	
PiB-NAV (n = 35)	3.1 ± 1.5
PiB-PiB (n = 27)	3.3 ± 1.7
**Cognitive status**	
Cognitively unimpaired	32
Cognitively impaired	3

PiB-PiB refers to a participant’s last two consecutive [^11^C]PiB observations while PiB-NAV refers to a participant’s last [^11^C]PiB observation and the following [^18^F]NAV4694 observation.

**Fig. 1.**
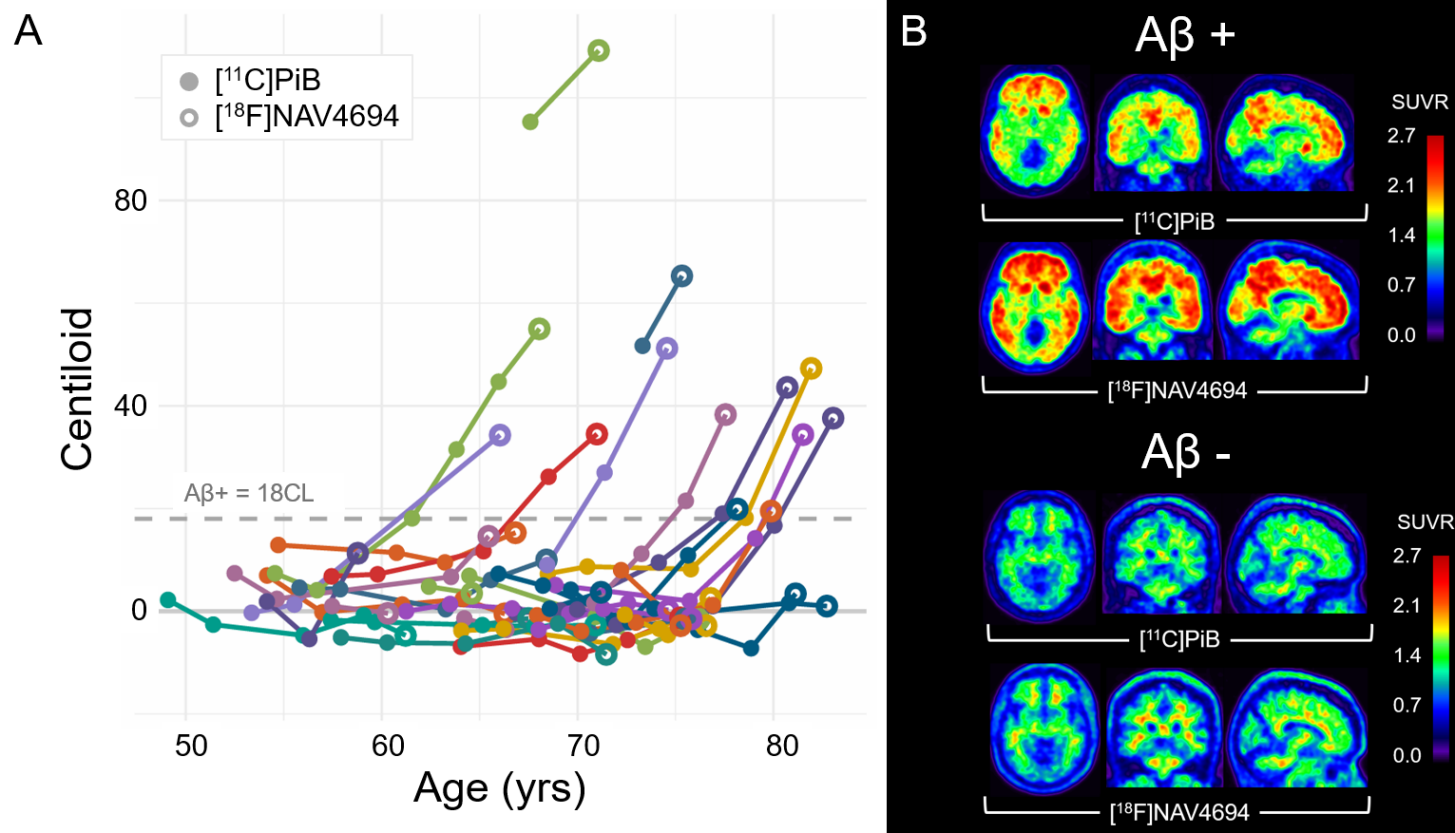
Longitudinal measures of CL values including 35 participants and 138 total amyloid PET scans (A). Amyloid burden measured with the terminal [^18^F]NAV4694 scan was consistent with the neuropathology time course as projected by prior [^11^C]PiB scans. [^11^C]PiB and [^18^F]NAV4694 images of an amyloid-positive and amyloid-negative participant in the cohort (B). Duration between scans was 3.5 and 2.9 years for the Aβ+ and Aβ- participants depicted.

Examples of both [^11^C]PiB and [^18^F]NAV4694 in an amyloid-negative and amyloid-positive participant are shown (Fig. 1). Higher extracerebral binding was observed in [^18^F]NAV4694 images relative to [^11^C]PiB. Longitudinal measures of CL values with respect to age are displayed in Figure 1.

### 3.2 Aβ- group

**Fig. 2.**
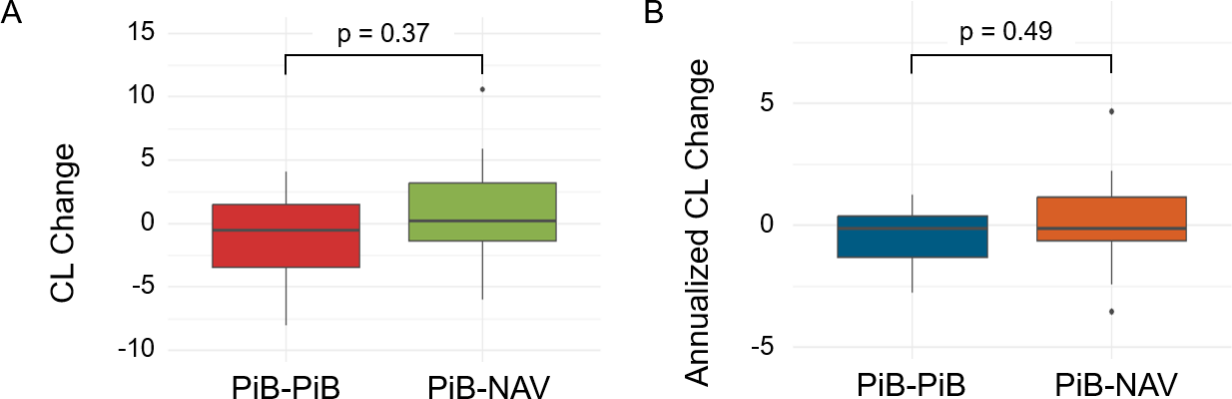
Box plots of CL change (A) and annualized CL change (B) in the amyloid-negative subset for all PiB-PiB and PiB-NAV observations. The PiB-PiB group includes 30 paired observations and PiB-NAV includes 18 paired observations.

The amyloid-negative subset consisted of 18 participants with 1 or more [^11^C]PiB scans followed by an [^18^F]NAV4694 scan (CL < 10). A Wilcoxon sum-rank test for raw CL change and annualized CL change showed no significant difference between consecutive [^11^C]PiB observations and the PiB-NAV observations for CL difference (p = 0.37) nor CL rate (p = 0.49) (Fig. 2). In 12 participants with more than 1 [^11^C]PiB scan followed by a [^18^F]NAV4694 scan (CL < 10), a Wilcoxon signed-rank test for raw CL change and annualized CL change showed no significant difference between the last consecutive [^11^C]PiB observations and the PiB-NAV observations for CL difference (p = 0.85) and CL rate (p = 0.97). Furthermore, a Friedman test was conducted on the 10 participants who had 3 or more [^11^C]PiB scans assessing CL difference and rate between PiB_t=1_-PiB_t=2_, PiB_t=2_-PiB_t=3_, and PiB_t=3_-NAV. No statistically significant differences emerged (CL difference (p =0.12), CL rate (p = 0.27)). [^18^F]NAV4694 SUVr was converted to [^11^C]PiB-equivalent [^18^F]NAV4694 (Rowe et al., 2016). Test–retest variability between radiotracers, defined as the absolute percentage difference between the [^11^C]PiB-equivalent [^18^F]NAV4694 SUVR and preceding [^11^C]PiB SUVr, was about 3.9% ± 3.3%, which lies within the typical test–retest variability reported for [^11^C]PiB (Lopresti et al., 2005; Okada et al., 2020; Tolboom et al., 2009). The linear mixed-effects model indicated that neither age, radiotracer, nor scanner significantly predicted CL in the amyloid-negative individuals (p > 0.05).

### 3.3 Aβ+ group

**Fig. 3.**
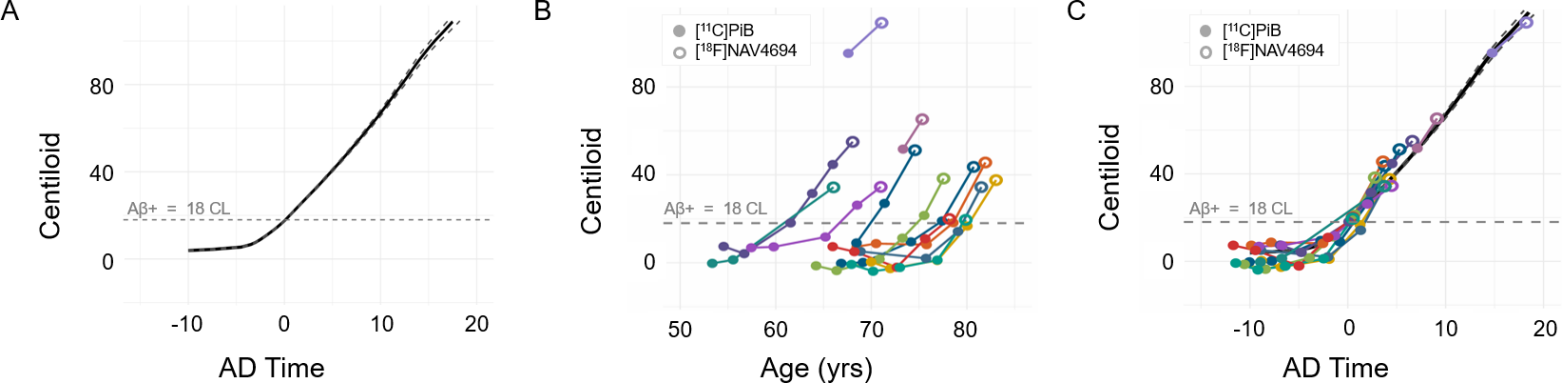
(A) SILA model generated with 172 [^11^C]PiB participants. (B) Age vs CL value relationship in Aβ+ [^18^F]NAV4694 participants. (C) Aβ+ participants aligned to the SILA model.

In target regions, all amyloid-positive scans monotonically increased in uptake, consistent with findings in the literature that Aβ continues to increase once Aβ + status is observed and at early disease stages (Landau et al., 2015). A total of 172 [^11^C]PiB participants from the WRAP study were used to generate an SILA model with predicted CL values up to an amyloid age of 24 years. This model was based upon previously published work within this cohort (Betthauser et al., 2022). A continuous and predictable increase in amyloid levels is exhibited from an amyloid age of 0–15 years. The [^11^C]PiB SILA CL rate from an amyloid age of 0–15 years is 5.2 ± 1.8 (mean ± SD) CL/year. The average rate of accumulation from the last [^11^C]PiB scan to the proceeding [^18^F]NAV4694 scan in the 13 amyloid-positive individuals was 6.0 ± 1.9 (mean ± SD) CL/year. Participants were aligned to the [^11^C]PiB generated SILA curve by their first positive scan shown below (Fig. 3). Amyloid burden indicated by [^18^F]NAV4694 was consistent with the disease timeline as projected with [^11^C]PiB.

### 3.4 Use of dual radiotracers

**Fig. 4.**
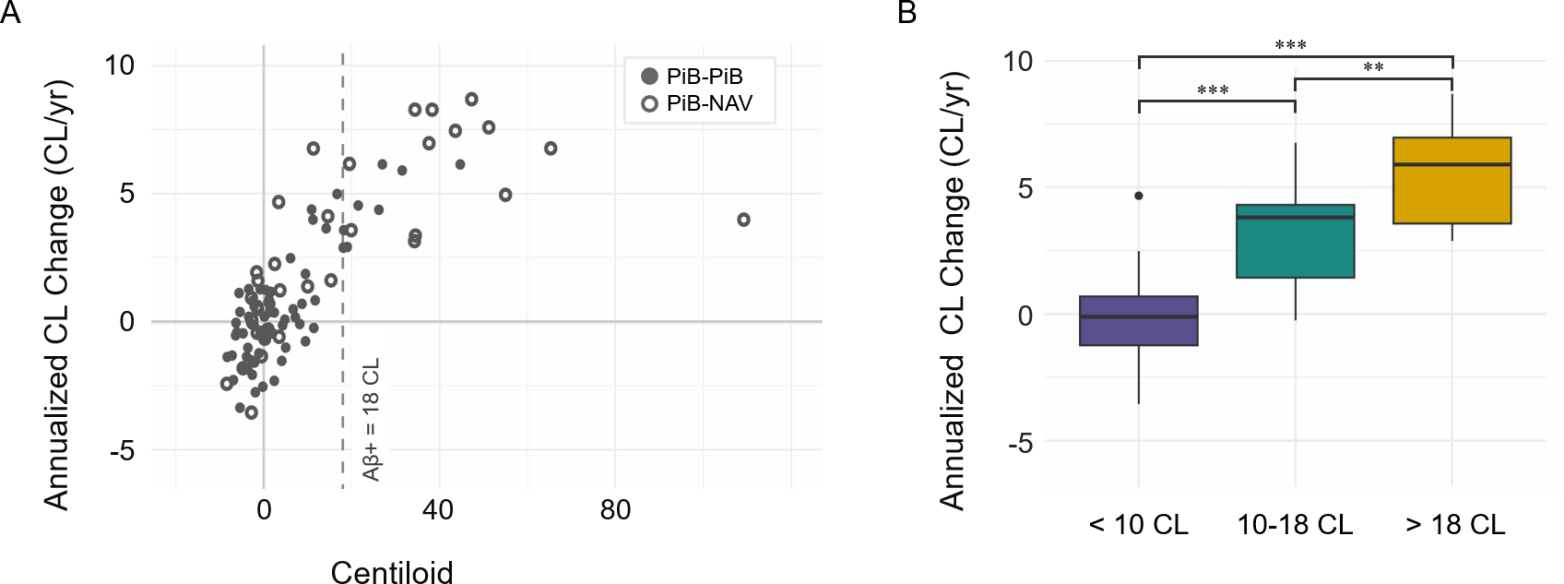
Amyloid accumulation rates including both PiB-to-PiB and PiB-to-NAV observations. Centiloid vs annualized rate is plotted based on the follow-up CL (A). Box plots of annualized rate in Aβ- and Aβ+ participants (B). Significance codes: ***p < 0.001, **p < 0.01.

To demonstrate the effect of transitioning between radiotracers in a longitudinal study, annualized change across all observations, PiB-PiB and PiB-NAV, was plotted as a function of CL value. The relationship between CL value and annualized rate upheld uniformity between PiB-PiB and PiB-NAV observations (Fig. 4). CL accumulation increased progressively across the defined CL thresholds (CL < 10, 10 < CL < 18, CL > 18), with average rates of -0.2 CL/year (95% CI [-0.53, 0.13]) for CL < 10, 3.1 CL/year (95% CI [1.6, 4.7]) for 18 > CL > 10, 5.5 CL/year (95% CI [4.6, 6.4]) for CL > 18.

## 4 Discussion

An optimal longitudinal PET imaging study design would employ a single Aβ-radiotracer and scanning conditions; however, practical considerations may require a transition between radiotracers. Although the Centiloid scale provides a standard method for harmonizing Aβ PET measures across radiotracers, differences in tracer imaging characteristics, such as white matter uptake, target affinity, and nonspecific binding, can require radiotracer-specific modifications to the standard image processing pipeline and other statistical adjustments. The goal of this study was to carefully examine the stability of Aβ burden outcome measures and the consistency of Aβ accumulation rates across two commonly used Aβ PET radiotracers. This work demonstrates that a uniform processing pipeline yields a continuous transition between [¹¹C]PiB and [^18^F]NAV4694, supporting their integration into longitudinal analyses.

[^18^F]NAV4694 has demonstrated nearly identical imaging characteristics to [^11^C]PiB. Prior validation work reported a strong correlation between [^11^C]PiB and [^18^F]NAV4694 SUVR ([^18^F]NAV4694_SUVR_ = 1.09[^11^C]PiB_SUVR_ – 0.08, R=0.99) (Rowe et al., 2016). Head-to-head evaluation of [^11^C]PiB and [^18^F]NAV4694 in post-mortem human brain tissue has also demonstrated a close similarity between regional binding, even finding a slightly higher affinity for Aβ plaque in [^18^F]NAV4694 than [^11^C]PiB (Aliaga et al., 2025). While previous longitudinal studies using [^18^F]NAV4694 have shown that this radiotracer can capture longitudinal increases in amyloid PET burden, our investigation directly demonstrates that [^18^F]NAV4694 tracks long-term amyloid accumulation with close fidelity to projections based on [11C]PiB data alone (Therriault et al., 2023; Zammit, Price, et al., 2025). In conducting these analyses, Aβ+ and Aβ- participants were analyzed separately. Aβ- individuals were evaluated for longitudinal stability, while Aβ+ individuals were evaluated for the rate of PiB-NAV change against a larger data-driven model of [^11^C]PiB accumulation. The SILA model and overlay of [^18^F]NAV4694 Aβ+ participants were included to illustrate the visual concordance between the population-based trajectory and individual observations. More specifically, the model reveals an expectation for the terminal [^18^F]NAV4694 observation if it was acquired with [^11^C]PiB. The PiB-NAV rate of Aβ accumulation appears higher (see Fig. 3) than that of the [^11^C]PiB SILA model where several terminal [^18^F]NAV4694 observations diverge from the SILA curve. Future investigations with a larger sample size will further clarify whether the difference reflects a true effect. One individual with profoundly elevated Aβ exhibited only moderate PiB-NAV amyloid increases. This observation may represent a plateau in amyloid deposition, wherein the rate of accumulation diminishes as the carrying capacity is approached (Jack, Wiste, et al., 2013). Statistical tests in the amyloid-negative subset found no differences in measured amyloid burden between radiotracers. The findings of this work reflect a high level of agreement between [^11^C]PiB and [^18^F]NAV4694 in derived outcome measures.

Longitudinal PET scans have demonstrated considerably higher measurement variability with other [^18^F]-amyloid radiotracers relative to [^11^C]PiB (Su et al., 2019). Several groups have attempted to reduce this variability with the use of a nonstandard reference region, such as a white matter reference region for [^18^F]florbetapir (Chen et al., 2015; Landau et al., 2015). Other studies have addressed longitudinal variability through image-driven methods, which have improved longitudinal consistency across amyloid radiotracers (Bourgeat et al., 2021, 2022). While our dataset spanned different scanners and radiotracers, we did not find appreciable differences in amyloid estimates across these technical factors. The consistency across radiotracers likely reflects the structural similarities between [^11^C]PiB and [^18^F]NAV4694, which create a uniquely suited pair of Aβ-radiotracers allowing for interchangeability that is unmatched by other [^18^F]-amyloid radiotracer pairings.

It is acknowledged that a more rigorous study design would acquire both [^11^C]PiB and [^18^F]NAV4694 scans at each time point to permit head-to-head cross-sectional and longitudinal validation simultaneously. A retrospective study design was chosen to minimize participant burden and radiation exposure and to leverage a large existing dataset. It is also important to note that, whereas prior [^11^C]PiB scans have subsequent PET-Aβ imaging to inform the disease timeline of the participant, the final [^18^F]NAV4694 scans lack follow-up observations to verify Aβ status. Consequently, amyloid-negative scans may express a small accumulation of amyloid that cannot definitively be attributed as either natural intra-individual variability or a true increase in amyloid burden. This condition suggests a potential bias in [^18^F]NAV4694 CL measurement toward a slight overestimation of amyloid within this subset. To help avoid capturing low levels of amyloid accumulation in early disease progression, a conservative threshold of 10 CL was imposed on the terminal [^18^F]NAV4694 observation. Though PiB-NAV CL difference and rate were descriptively higher in the box plots, PiB-NAV and PiB-PiB groups were not statistically different.

This study investigated a direct longitudinal comparison of PiB-to-PiB vs PiB-to-NAV amyloid accumulation. [^18^F]NAV4694 demonstrated both consistency in trending amyloid accumulation and constancy in sustained amyloid-negative participants compared with [^11^C]PiB. These results highlight how both radiotracers [^11^C]PiB and [^18^F]NAV can be integrated into a single analytical framework under the standard CL processing pipeline.

## Data Availability

PET imaging data from the University of Wisconsin—Madison Alzheimer’s Disease Research Center can be requested using an online application process (https://www.adrc.wisc.edu/apply-resources).
